# A Review of the Effects of Fucoxanthin on NAFLD

**DOI:** 10.3390/nu15081954

**Published:** 2023-04-19

**Authors:** Nor Hafiza Sayuti, Khairul Najmi Muhammad Nawawi, Jo Aan Goon, Norfilza Mohd Mokhtar, Suzana Makpol, Jen Kit Tan

**Affiliations:** 1 Department of Biochemistry, Faculty of Medicine, Universiti Kebangsaan Malaysia, Bandar Tun Razak, Cheras, Kuala Lumpur 56000, Malaysia; 2 Gastroenterology and Hepatology Unit, Department of Medicine, Faculty of Medicine, Universiti Kebangsaan Malaysia, Bandar Tun Razak, Cheras, Kuala Lumpur 56000, Malaysia; 3 GUT Research Group, Faculty of Medicine, Universiti Kebangsaan Malaysia, Bandar Tun Razak, Cheras, Kuala Lumpur 56000, Malaysia; 4 Department of Physiology, Faculty of Medicine, Universiti Kebangsaan Malaysia, Bandar Tun Razak, Cheras, Kuala Lumpur 56000, Malaysia

**Keywords:** fucoxanthin, NAFLD, non-alcoholic fatty liver disease, steatosis, lipid metabolism

## Abstract

Non-alcoholic fatty liver disease (NAFLD) is the most prevalent form of chronic liver disease. Fucoxanthin, a red-orange marine carotenoid, is found in natural marine seaweeds with high antioxidant activity and several other remarkable biological features. The aim of this review is to gather evidence of the positive benefits of fucoxanthin on NAFLD. Fucoxanthin provides an extensive list of physiological and biological properties, such as hepatoprotective, anti-obesity, anti-tumor, and anti-diabetes properties, in addition to antioxidant and anti-inflammatory properties. This review focuses on published research on the preventative effects of fucoxanthin on NAFLD from the perspective of human clinical trials, animal experiments *in vivo*, and *in vitro* cell investigations. Using a variety of experimental designs, including treatment dosage, experiment model, and experimental periods, the positive effects of fucoxanthin were demonstrated. Fucoxanthin’s biological activities were outlined, with an emphasis on its therapeutic efficacy in NAFLD. Fucoxanthin showed beneficial effects in modulating lipid metabolism, lipogenesis, fatty acid oxidation, adipogenesis, and oxidative stress on NAFLD. A deeper comprehension of NAFLD pathogenesis is essential for the development of novel and effective therapeutic strategies.

## 1. Introduction

Non-alcoholic fatty liver disease (NAFLD) is a global epidemic and a major contributor to the risk of dying from metabolic and liver disorders. It affects about 25% of the global population, with increasing prevalence in individuals with metabolic diseases (e.g., 50.7% of overweight/obese adults were diagnosed with fatty liver) [1]. NAFLD includes a broad range of pathological disorders, from mild steatosis to steatohepatitis (NASH), severe fibrosis, cirrhosis, and hepatocellular cancer, but has no recognized drug treatment [2,3]. NAFLD is associated with metabolic syndrome including obesity [4,5], insulin resistance [6,7], hypertension [8,9], dyslipidemia [10,11], cardiovascular diseases [12], and type 1 or type 2 diabetes [6,13,14]. Hepatic steatosis is a defining feature of NAFLD in the absence of any other definite causes, such as hepatic viral infection, drug-induced hepatotoxicity, excessive alcohol use, or hereditary factors [15]. An inconsistency between lipid availability and lipid elimination leads to lipid build-up in the liver. These hazardous lipid species lead to fibrogenesis and genetic instability by inducing hepatocellular stress, damage, and death, which results from the dynamic interaction of environmental elements (such as dietary patterns), obesity, modifications in the microbiota, and susceptibility genetic variations [16,17]. The term metabolic dysfunction-associated fatty liver disease (MAFLD), which better describes the pathophysiology of this liver disease and the associated metabolic abnormalities, was proposed in 2020 as a replacement for the term NAFLD [18]. Unfortunately, the terminology change also resulted in the diagnostic criteria changing. NAFLD is defined as the presence of fatty liver disease in the absence of established causes of steatosis. On the other hand, MAFLD is characterized as the existence of fatty liver disease along with the presence of excess weight, obesity, and/or type 2 diabetes mellitus [19]. The adoption controversy has been fueled by a few editorials and opinion articles that have been published [20,21,22,23,24].

Pharmaceutical treatment for NAFLD has not yet received approval from the Food and Drug Administration (FDA) [25]. There are currently no effective treatments for NAFLD; only weight loss has been observed to ameliorate the histological characteristics of NAFLD [26,27]. Owing to NAFLD’s increasing incidence and few available treatment options, it is necessary to discover new active compounds with promising preventative and/or therapeutic effects on NAFLD.

Fucoxanthin is a marine carotenoid belonging to the xanthophyll family with an allenic bond, a conjugated carbonyl, a 5,6-monoepoxide, and an acetyl group within its structure. Willstatter and Page isolated this compound from brown algae in 1914 [28]. Fucoxanthin is commonly present in edible brown algae, including dashima (*Laminaria japonica*), wakame (*Undaria pinnatifida*), hijiki (*Hizikia fusiformis*), and gulfweed (*Sargassum fulvellum*) [29]. Several non-allenic carotenoids, including β-carotene 5,6-epoxide, lutein, and lutein epoxide, did not suppress fat build-up in adipose tissue or liver, suggesting that the allene bond in fucoxanthin and its derivatives may be required for fat deposition inhibition [30]. Furthermore, fucoxanthin also has been reported and acknowledged for its health benefits such as in the prevention of cancer [31], anti-obesity [32], immunomodulation of inflammation, and antioxidant activity [33]. Fucoxanthin possesses powerful anti-inflammatory [34] and cancer-preventative properties due to its antioxidant properties [35].

The goal of this review is to emphasize the effects of fucoxanthin on NAFLD and the underlying mechanisms which have been explored by published studies in preclinical and clinical research, as well as the potential processes behind these effects.

## 2. Effect and Mechanisms of Fucoxanthin on NAFLD

### 2.1. Clinical Trials

There are very few published analyses of the effects of fucoxanthin on NAFLD patients. To our knowledge, only three human studies investigating the effects of fucoxanthin on NAFLD have been reported to date (Table 1). Shih et al. [35] conducted a randomized placebo-controlled clinical trial of 42 NAFLD patients with a 24-week follow-up period. Three capsules of low-molecular-weight fucoidan and high-stability fucoxanthin (LMF-HSFx) were administered twice daily to patients in the treatment group. The ALT/AST (alanine aminotransferase/aspartate aminotransferase) ratio has been proposed as an alternate diagnostic for hepatic steatosis [36]. Serum AST/ALT levels dropped in the LMF-HSFx treatment group compared to the placebo group after 6 months, indicating a reduction in hepatic lipotoxicity in NAFLD patients. Additionally, LMF-HSFx lowers fibrosis and hepatic steatosis in NAFLD patients with a decrease in the relative ratio of controlled attenuation parameter (CAP) value and stiffness degree to baseline (%) compared to the placebo. The expression of cytokines interleukin (IL)-6 and interferon (IFN)-γ was downregulated at the third and sixth months in the LMF-HSFx group compared to the placebo, thus reducing inflammation in patients with NAFLD. Furthermore, LMF-HSFx modulates adipogenesis in the LMF-HSFx group, increasing adiponectin and leptin levels relative to the placebo. For adipokines, relative ratios to baseline (%) of leptin were increased compared to the placebo, while relative ratios to baseline (%) of adiponectin were not statistically different from the placebo. LMF-HSFx demonstrated the ability to decrease insulin resistance and improve β-cell repair in patients with NAFLD. In metabolic terms, insulin resistance shows the inability of a specific amount of insulin to metabolize a known glucose level in an individual compared to the population as a whole [37]. Insulin inhibits lipolysis, regulates triglyceride (TG) deposition in adipose tissue, and increases fatty acid esterification and storage. As a result, insulin resistance becomes a critical treatment element in NAFLD [38]. However, the understanding of the long-term application may be constrained by the relatively shorter period of observation in this study. To reach a definitive conclusion, the author stated that future research should involve a larger sample size of patients and long-term follow-up evaluations [35].

Alternatively, a randomized double-blind placebo-controlled trial utilizing LMF-HSFx on NAFLD patients was also reported by Cheng et al. [39]. The experiment design was outlined in Table 1 (second row). After 1 and 3 months of treatment, there was a reduction in serum ALT in the LMF-HSFx group compared to the control group, suggesting that LMF-HSFx supplementation provided hepatoprotective activity and reduced liver injury. The supplement did not have an anti-steatosis impact on NAFLD patients. On a clinical basis, NAFLD begins as simple steatosis (NAFL). The excessive amount of liver fat causes inflammation by invading immune cells and secreting cytokines. This is known as non-alcoholic steatohepatitis, or NASH, and it can lead to liver fibrosis [40]. This study [39] did not detect any significant difference in CAP, adiponectin, and the severity of fibrosis; thus, further research was required. The authors concluded that this combination could be a potential hepatoprotective supplement for NAFLD patients [39].

Abidov and colleagues [41] examined the relationship of Xanthigen (brown marine algae fucoxanthin + pomegranate seed oil) consumption in women with NAFLD and women with normal liver fat (NLF). In obese women without diabetes, Xanthigen caused a reduction in body weight, and body and liver fat content. Moreover, the plasma levels of the enzymes ALT, AST, and γ-glutamyltransferase (GGT) were all reduced by Xanthigen compared to the placebo. Additionally, consuming Xanthigen and fucoxanthin enhanced the levels of resting energy expenditure (REE). This product has the potential to be a good weight loss supplement. Due to the small number of participants, this portion of the study is exploratory in nature. These results would need to be confirmed by a larger sample size [41].

**Table 1:** Effects of fucoxanthin on NAFLD in human trials.

Human Trial and Duration of Experiment	Experimental Groups	Effects	Mechanisms	References
42 NAFLD patients 24 weeks	Control group: 3 capsules of 550 mg/capsule cellulose powder Treatment group: 3 capsules of LMF-HSFx (each capsule contains 275 mg LMF and 275 mg HSFx) twice/day	↓ Hepatic lipotoxicity ↓ Hepatic steatosis ↓ Fibrosis ↓ Insulin resistance ↓ TC ↓ TG	↓ IL-6 ↓ IFN-γ	Shih et al., 2021 [35]
42 NAFLD patients 12 weeks	Control group: Placebo; Treatment: 3 capsules of LMF-HSFx (each capsule contains 275 mg LMF and 275 mg HSFx) twice/day	↓ ALT ↓ Liver injury No effect on liver steatosis	-	Cheng et al., 2019 [39]
113 NAFLD and 38 NLF patients 16 weeks	Placebo-NAFLD, Xanthigen-NAFLD, placebo-NLF, and Xanthigen-NLF group	↓ Body weight ↓ Body liver fat content ↓ Waist circumference ↓ CRP ↓ TG ↓ ALT ↓ AST ↓ GGT	-	Abidov et al., 2010 [41]

↓: down-regulation; ALT: alanine aminotransferase; AST: aspartate aminotransferase; CRP: C-reactive protein; IFN-γ: interferon-γ; IL-6: interleukin-6; LMF-HSFx: low-molecular-weight fucoidan and high-stability fucoxanthin; GGT: γ-glutamyltransferase; NLF: normal liver fat; TC: total cholesterol; TG: triglyceride.

### 2.2. Animal Studies

To date, five animal studies on the potential effect of fucoxanthin on NAFLD have been identified, and their findings are summarized in Table 2. Kim et al. [42] conducted their first study (12 weeks) with male C57BL/6J mice that consumed either a high fat, high sugar, and high cholesterol (HFC) diet alone, or an HFC diet supplemented with 0.015% or 0.03% (*w*/*w*) fucoxanthin powder. A diet-induced obesity (DIO) mouse model was used to develop non-alcoholic steatohepatitis (NASH), a more advanced stage of NAFLD. In the second (8-week) study [42], the mice consumed either a high fat, high sucrose (HFS) diet or an HFS diet containing 0.01% fucoxanthin powder. Treatment with fucoxanthin did not cause the obese mice that were fed an HFC or HFS diet to lose weight after feeding the period time. The results also show no statistically significant changes between the control group and the groups that were fed either HFC or HFS in terms of total cholesterol (TC) and liver TG levels, hepatic fat build-up, or serum ALT levels. Nevertheless, fucoxanthin increased the expressions of mRNA which regulate the biogenesis of mitochondrial and fatty acid oxidation in the soleus muscle, while simultaneously increasing the number of mitochondrial DNA copies relative to the HFC group. All fucoxanthin treatment groups showed significantly higher hepatic expression of genes involved in the lipogenic process, including fatty acid synthase (FAS) and sterol regulatory element-binding protein factor 1c (Srebf1) than the HFC or HFS control groups. Compared to the HFC control group, fucoxanthin significantly increased the hepatic expression of genes involved in cholesterol metabolism, such as hydroxy-3-methylglutaryl-coenzyme A reductase (Hmgcr) and low-density lipoprotein receptor (Ldlr). Fucoxanthin also increased the hepatic fatty acid β-oxidation genes (acyl-coenzyme A oxidase 1 (Acox1)) in the 0.03% fucoxanthin group relative to the HFC group. According to the authors, it is unknown what caused this discrepancy between their study’s results and those of previous studies, but it can result from variations in mice strains, food content, the duration of the diet plan, fucoxanthin dose, and other dietary components used in dietary treatments. The authors summarized and concluded that fucoxanthin intake had minimal effects on hepatocytes, and white adipose fibrosis and inflammation in two distinct DIO animal models after 12 weeks or 8 weeks of feeding [42].

Shih et al. [35] also explored how LMF-HSFx affected hepatic lipotoxicity in male C57BL/6 mice for 16 weeks. The mice were put into six groups at random, as described in Table 2. The level of fasting blood glucose increased in the HFD mice compared to the ND group, whereas 400 mg/kg/BW/day of LMF-HSFx therapy lowered the serum glucose level in the HFD mice. TG content was also lower in the HFD mice supplemented with the LMF-HSFx group compared to the HFD group, whereas the serum AST and ALT levels were higher in the HFD group compared to the ND group, thus indicating that LMF-HSFx significantly reduced the lipotoxicity caused by HFD in the liver. Lipotoxicity is induced by prolonged elevations of free fatty acids (FFAs) in non-adipose cells and is thought to have a role in the development of NAFLD. In addition, the adiponectin expression genes adipoq and adig, as well as the leptin expression gene lep, were upregulated in the LMF-HSFx group, suggesting that LMF-HSFx can control adipogenesis in the mice that were fed an HFD [35]. As NAFLD progresses, TGs increase within hepatocytes, causing typical vacuoles that resemble adipocytes. Pathogenesis of NAFLD is augmented by defective adipose tissue, which increases the supply of fats and adipokines to the liver, causing hepatic steatosis and inflammation. As a result, defective adipose-liver crosstalk plays an important role in the development and progression of NAFLD [43].

Fucoxanthin’s ability to inhibit the variables linked to the development of NASH was studied by Takatani et al. in 2020 [44]. Untreated NASH can progress to more acute liver conditions such as hepatocellular carcinoma and cirrhosis, with a higher risk of development of hepatocellular carcinoma. Table 2 outlines the experimental design and experimental group (third row). Following 4 weeks of experimental feeding, fucoxanthin reduced liver damage in the diet-induced NASH mice model by lowering hepatic fat build-up and lipid oxidation. The serum levels of AST and ALT were lower in the fucoxanthin group compared to the CDAHFD group. In addition, the total lipid, TG, and TC levels were lower in the fucoxanthin group when compared to the CDAHFD group. Fucoxanthin’s suppressive effect on lipid oxidation was demonstrated by the lower levels of hepatic thiobarbituric acid-reactive substances (TBARS) and lipid hydroperoxide in the CDAHFD-fed mice. Furthermore, when compared to the diet-induced NASH model mice, fucoxanthin also helps to reduce inflammation and the development of fibrosis. In comparison to the CDAHFD group, fucoxanthin reduced the levels of inflammatory cytokines and chemokines including Tnfα, IL-6, IL-1b, and Mcp-1. Fucoxanthin significantly downregulated the mRNA expression of fibrogenic factors including Tgfβ1, Col1α1, Timp1, and the activated-HSC marker αSMA compared to the CDAHFD group. The authors concluded that in the diet-induced NASH model mice, dietary fucoxanthin protected early fibrosis by reducing hepatic fat formation, oxidative stress, and inflammation.

Ha and Kim [30] conducted an *in vivo* study for 4 weeks using 0.2% fucoxanthin as a nutritional supplement in HFD rats (HF group). In this research, the consumption of fucoxanthin reduces the accumulation of lipids in the livers of HF rats. Total hepatic lipid was reduced in the HF + Fxn group compared to the HF group. The HF + Fxn group had lower levels of hepatic TC and TG than the HF group. In addition, the HF + Fxn group had higher fecal TC levels compared to the HF group. Fucoxanthin supplementation increased the concentration of fecal cholesterol and total lipids in the HF group. In addition, the consumption of this compound suppressed the gene expression of transcription regulators and enzymatic activities in hepatic lipogenesis including FAS, acetyl-CoA carboxylase (ACC), and glucose-6-phosphate dehydrogenase (G6PDH) compared to the HF group. Furthermore, the fucoxanthin intake lowered the mRNA expression of acyl-CoA cholesterol acyltransferase (ACAT) and hydroxy-3-methylglutaryl coenzyme A (HMG-CoA) reductase compared to the HF group. The consumption of fucoxanthin is believed to improve cholesterol and lipid metabolisms in HF rats [30].

Park et al. [45] investigated the effect of fucoxanthin-rich extracts (UEFx) extracted from dried brown algae (*Undaria pinnatifida*) and pure fucoxanthin on male C57BL/6J mice that were fed a high fat control diet (HC group) for 9 weeks. Supplementation with UEFx or pure fucoxanthin managed to prevent hepatic fat accumulation and insulin resistance in the HC mice. UEFx prevented insulin resistance and the buildup of hepatic fat in the HC-induced obese mice; this effect is partially mediated by regulating hepatic glucose and lipid balance [45].

**Table 2:** Effects of fucoxanthin on NAFLD in animal studies.

Animal Model and Duration of Experiment	Experimental Groups	Effects	Mechanisms	References
Male C57BL/6J Mice 8 or 12 weeks	5 groups 12 weeks: High fat/high sucrose/high cholesterol (HFC) diet, 34% sucrose, 2.0% cholesterol, 34% fat; HFC + 0.015% fucoxanthin, HFC + 0.03% fucoxanthin 8 weeks: High fat/high sucrose (HFS) diet, 34% fat, 35% sucrose; HFS + 0.01% fucoxanthin	HFC + 0.015% or 0.03% fucoxanthin: ↑ BW ↑ Serum TC ↑ Mitochondrial DNA HFS + 0.01% fucoxanthin: ↑ BW ↑ Serum TC ↑ Liver TG	HFC + 0.015% or 0.03% fucoxanthin: ↑ Srebf1 ↑ FAS ↑ Ldlr ↑ Hmgcr ↑ Tfam ↑ Esrrα ↑ Ppargc1a ↑ Acox1 (0.03% fucoxanthin only) HFS + 0.01% fucoxanthin: ↑ Srebf1 ↑ FAS ↑ Ucp2 ↑ Acox1	Kim et al., 2022 [42]
Male C57BL/6 mice 16 weeks	6 groups Normal diet (ND): 200 g of casein and 70 g of soybean oil/kg; ND + 200 mg/kg/BW/day of LMF-HSFx; ND + 400 mg/kg/BW/day of LMF-HSFx; High fat diet (HFD): ND + 60% fat-derived calorie; HFD + 200 mg/kg/BW/day of LMF-HSFx; HFD + 400 mg/kg/BW/day of LMF-HSFx	↓ Hepatic lipotoxicity ↓ Lipid droplet ↑ Adipogenesis ↓ Blood glucose ↓ TG ↓ TC ↓ AST ↓ ALT	↑ Adipoq ↑ Adig ↑ Lep	Shih et al., 2021 [35]
Male C57BL/6J Mice 4 weeks	3 groups Control diet (Research Diet, Inc. A06071314); Choline-deficient L-amino acid-defined high fat diet (CDAHFD); CDAHFD + 0.2% fucoxanthin	↓ BW ↓ Liver weight gain ↓ Total lipid ↓ TG ↓ TC ↓ AST ↓ ALT ↓ TBARS ↓ Lipid hydroperoxide ↓ Lipid droplets ↓ Fibrosis ↑ Lipid metabolism ↓ Oxidative stress ↓ Hepatic fat accumulation ↓ Biomakers of inflammation and infiltration	↓ Tnfα ↓ Il-6 ↓ Il-1b ↓ Mcp-1 ↓ Tgfβ1 ↓ Col1α1 ↑ Timp1 ↓ F4/80 ↓ Cd11c ↓ Ccr2 ↓ Ly6c ↓ αSMA	Takatani et al., 2020 [44]
Male Sprague-Dawley Rat 4 weeks	3 groups Normal diet + 7% fat (soybean oil); High fat diet + 20% fat (13% lard, 7% soybean oil) (HF); HF + 0.2% fucoxanthin diet (HF + Fxn)	↑ Plasma HDL ↓ Hepatic total lipids ↓ Hepatic cholesterols ↓ Hepatic triglycerides ↑ Fecal triglyceride ↑ Fecal cholesterol ↑ Fecal total lipids	↓ ACC ↓ FAS ↓ G6PDH ↓ SREBP-1c ↑ CPT1 ↑ CYP7A1 ↓ HMG-CoA ↓ ACAT ↑ LCAT	Ha and Kim, 2013 [30]
Male C57BL/6J Mice 9 weeks	4 groups Normal diet; High fat control diet (3% corn oil and 17% lard) (HC); HC + 0.02% fucoxanthin; HC + 0.69% *U. pinnatifida* ethanol extract (UEFx)	↓ Visceral fat ↓ Adipocyte size ↓ Fasting blood glucose ↓ Plasma insulin ↓ Insulin resistance index ↑ β-oxidation ↑ Glycolytic enzyme ↓ Hepatic lipid accumulation ↑ Glycogen content	↓ PEPCK ↓ PAP ↑ GK/G6Pase	Park et al., 2011 [45]

↑: up-regulation; ↓: down-regulation; ACC: acetyl-CoA carboxylase; ACAT: acyl-CoA cholesterol acyltransferase; Acox1: acyl-coenzyme A oxidase 1; ALT: alanine aminotransferase; AST: aspartate aminotransferase; BW: body weight; Ccr2: chain C–C chemokine receptor type 2; Cd11c: integrin alpha X chain protein; CPT1: carnitine palmitoyltransferase-1; Col1α1: collagen type I alpha 1; CYP7A1: cholesterol 7α-hydroxylase1; Esrrα: estrogen-related receptor α; FAS: fatty acid synthase; GK: glucokinase; G6Pase: glucose-6-phosphatase; G6PDH: glucose-6-phosphate dehydrogenase; HDL: high-density lipoprotein; HMG-CoA: hydroxy-3-methylglutaryl coenzyme A; Hmgcr: hydroxy-3-methylglutaryl-coenxyme A reductase; LCAT: lecithin–cholesterol acyltransferase; Ldlr: low-density lipoprotein receptor; LMF-HSFx: low-molecular-weight fucoidan and high-stability fucoxanthin; Mcp-1: monocyte chemoattractant protein-1; PAP: phosphatidate phosphohydrolase; PEPCK: phosphoenolpyruvate carboxykinase; Ppargc1a: peroxisome proliferator-activated receptor-γ coactivator 1α; Srebf1: sterol regulatory element-binding protein factor 1c; TBARS: thiobarbituric acid-reactive substances; Timp1: tissue inhibitor matrix metalloproteinase 1; TC: total cholesterol; TG: triglycerides; Tgfβ1: pro-fibrogenic cytokine transforming growth factor beta 1; Tfam: mitochondria transcription factor A; Ucp2: uncoupling protein 2.

### 2.3. In Vitro Studies

Table 3 summarizes the results of four *in vitro* cell experiments on the possible impact of fucoxanthin on NAFLD that have been conducted so far. Ye et al. [46] used normal human Chang liver cells to investigate the effects and mechanisms of fucoxanthin in the FFA-induced NAFLD cell model. The fucoxanthin high-dose (H-Fx) group results demonstrated a rise in superoxide dismutase (SOD) expression and a decrease in the amount of interleukin-1 beta (IL-1β) compared to the FFA group, suggesting that fucoxanthin aids in lowering the levels of oxidative stress and inflammation caused by FFA in the liver cells. The protrusions were visible, and there were fewer lipid accumulations in the cytoplasm of cells that had been treated with fucoxanthin. Fucoxanthin can also reverse FFA-induced NAFLD by upregulating the nuclear factor erythroid-2-related factor 2 (Nrf2) and adenosine monophosphate-activated protein kinase (AMPK) signaling pathways and inhibiting the expression of protein involved in the Toll-like receptor 4 (TLR4) signaling pathway. Fucoxanthin showed ameliorative advantages in the FFA-induced NAFLD cell model and might be researched as a promising anti-NAFLD agent.

Human HepaRG hepatocytes were used in a study performed by Shih et al. [35]. HepaRG cells were grown in the HepaRG Induction Medium, which was made up of bovine serum-conjugated palmitic acid (PA) with or without 25 µg/mL LMF-HSFx. Cells treated with LMF-HSFx had modified the SIRI-PGC-1 pathway by increasing the expression of SIRT2, 3, and 6 in PA-induced lipotoxicity in cells. The SIRI-PGC-1 pathway is acknowledged as one of the most intriguing NAFLD targets because it affects lipid metabolism, mitochondrial activation, and liver inflammation. After observing LMF-HSFx’s protective effects, the results showed that in PA-treated HepaRG cells, LMF-HSFx restored cell cycle arrest, decreased Caspase 3 activation, and increased mitochondrial integrity.

HPLC analysis conducted by Takatani et al. [44] found that the liver lipid extract contained the fucoxanthin metabolites amarouciaxanthin A (Amx A) and fucoxanthinol (FxOH). These two metabolites reduce inflammation in TNFα-stimulated Hepa1-6 cells by inhibiting chemokine secretion of monocyte chemoattractant protein-1 (Mcp-1) and downregulating the mRNA expression of chemokines such Mcp-1 and C-C motif chemokine ligand 5 (Ccl5).

Meanwhile, Chang et al. [47] examined the ability of fucoxanthin to prevent lipid accumulation in a fatty acid-induced FL83B cell model. Fucoxanthin decreased the expression of lipogenic-related transcription factor (sterol regulatory element-binding protein-1c (SREBP-1c), peroxisome proliferator-activated receptor-γ (PPAR-γ)), and FAS. Fucoxanthin showed promising therapeutic potential in oleic acid-induced fatty liver cells by improving the Sirt1/AMPK pathway, promoting lipolysis, and blocking lipogenesis. Moreover, fucoxanthin can lower hepatic lipid build-up by suppressing transcription factors of lipogenesis and promoting β-oxidation in hepatocytes.

**Table 3:** Effects of fucoxanthin on NAFLD in cell models.

*In Vitro* Model	Experimental Design	Effects	Mechanisms	References
Normal human Chang liver cells	NAFLD cells: 1 mM FFA mixture; oleic acid and palmitic acid (2:1, *v/v*) for 24 h Treatment: 0.125, 0.25, 0.5, 1, 2 and 8 μg/mL of fucoxanthin, incubated for another 24 h	↓ Oxidative stress ↓ Inflammatory levels ↓ Lipid droplets	↓ IL-1β ↑ SOD ↓ Keap-1 ↑ Nrf2 ↑ HO-1 ↑ NQO1 ↑ GCLM ↑ AMPK ↓ TLR4 ↓ MyD88 ↓ p-IκBα ↓ p-NF-κB p65 ↓ SREBP-1c ↓ FAS	Ye et al., 2022 [46]
Human HepaRG^TM^ cells	NAFLD cells: bovine serum-conjugated PA Treatment: 25 μg/mL LMF-HSFx, co-treated for 24 h	↓ Cell death ↓ DNA fragmentation ↑ Mitochondrial integrity	↑ SIRT2, 3, 6, ↑ PGC-1β ↑ ATGL	Shih et al., 2021 [35]
Hepa1-6 cells	TNFα-stimulated cells: carotenoids (2.5 mM) + TNFα (10 ng/mL) for 3 h (gene) or 24 h (for protein secretion)	Anti-inflammatory ↓ Chemokine production	↓ Mcp-1 ↓ Ccl5	Takatani et al., 2020 [44]
FL83B cells	NAFLD cells: 0.5 mM oleic acid for 48 h Treatment: 3–100 mM fucoxanthin, incubated for another 24 h	↓ Lipid accumulation ↓ Lipid peroxidation ↓ Lipogenesis	↓ SREBP-1c ↓ PPAR-γ ↑ AMPK ↓ ACC ↑ Sirt1/AMPK	Chang et al., 2018 [47]

↑: up-regulation; ↓: down-regulation; ACC: acetyl-CoA carboxylase; AMPK: AMP-activated protein kinase; ATGL: adipose triglyceride lipase; Ccl5: C-C motif chemokine ligand 5; FAS: fatty acid synthase; FFA: free fatty acids; GCLM: glutamate-cysteine ligase modifier subunit; HO-1: heme oxygenase-1; IL-1β: interleukin-1 beta; Keap-1: Kelch-like ECH-associated protein 1; LMF-HSFx: low molecular weight fucoidan and high-stability fucoxanthin; Mcp-1: monocyte chemoattractant protein-1; NQO1: NAD(P)H quinone dehydrogenase 1; MyD88: myeloid differentiation factor 88; Nrf2: nuclear factor erythroid 2–related factor 2; PA: palmitic acid; PGC-1β: proliferator-activated receptor-γ coactivator-1β; PPAR-γ: peroxisome proliferator-activated receptor-γ; p-IκBα: phosphorylated inhibitor of nuclear factor kappa B; SOD: superoxide dismutase; SIRT: sirtuin; SREBP-1c: sterol regulatory element-binding protein-1c; TLR4: toll-like receptor 4; *v*/*v*: volume/volume.

## 3. Limitation and Future Perspectives

According to research, fucoxanthin may be beneficial in preventing chronic illnesses such as cancer, diabetes, liver disease, and obesity. Fucoxanthin may be used therapeutically or as a preventative measure for NAFLD/MAFLD. Studies on animals indicate that fucoxanthin supplementation possesses some positive effects and has no negative side effects. Fucoxanthin and its metabolites have been shown to be safe, non-toxic, and easily administered [48]. No mortality or abnormalities were reported in rats administered with a single oral dose of 2000 mg/kg/BW/day of fucoxanthin or 20 or 200 mg/kg/BW/day of fucoxanthin for 13 weeks. Therefore, it appears safe to consume fucoxanthin, at least according to experiments conducted on animals [49,50,51]. However, human studies regarding the safety of fucoxanthin intake are scarce. Despite the fact that no toxic effects of fucoxanthin have been reported in animal studies, human clinical trials are required to determine its safety. Irrespective of the lack of clinical trials to evaluate its safety, the FDA has approved fucoxanthin extracted from the microalga as a new nutritional supplement that can be consumed at a dosage of 3 mg daily for an indefinite period or 5 mg fucoxanthin daily for up to 90 days [52]. However, human trials are required to examine the safety of fucoxanthin as well as to investigate the mechanism through which fucoxanthin exhibits its therapeutic benefits [53].

In clinical trials, it is crucial to have an understanding of the limits of clinical studies. It is possible that the duration of therapy is insufficient to uncover issues resulting from long-term histological alteration. The number of included randomized trials was also limited, as were the trial subjects. On the other hand, the use of oral formulations with varied dosages, which may not always assure optimal absorption in patients with liver disease, may further contribute to fucoxanthin’s inefficacy. In addition, because few studies have explored the mechanism of action behind the therapeutic effects of fucoxanthin in human trials, it is not possible to determine the optimal conditions for recognizing their positive effects.

Even though research has shown that fucoxanthin has various therapeutic and nutritional benefits, its structure is unstable. The two primary metabolites of fucoxanthin are Amx A and FxOH [54]. Furthermore, fucoxanthin can be metabolized to FxOH in the gastrointestinal tract and then to Amx A in the liver. The rate of fucoxanthin absorption may be greatly altered by some components, particularly lipids. Further study is also required to find new biological functions of fucoxanthin metabolites in the livers of patients with NAFLD that may be different from the parent compound. *In vitro* experiments with fucoxanthin derivatives are required to provide a deeper understanding of the mechanisms of how fucoxanthin mediates its health advantages [53]. Moreover, the majority of *in vivo* animal studies have been conducted on male mice/rats; therefore, the idea of sexual dimorphism has not been explored. To gain a greater understanding of the principles behind the effects of fucoxanthin in both sexes, further study with both male and female mice/rats is required.

Despite the high prevalence of NAFLD/MAFLD and the extensive research that has been carried out in the area, the treatment for it remains a challenge. It is widely acknowledged that patients with NAFLD/MAFLD should practice a healthier lifestyle including maintaining a healthy diet, working out frequently, and maintaining body weight. However, for some reason, the majority of individuals are unable to partake in such activities. Instead, more treatments are required to address NAFLD’s adverse effects [55]. There is currently little clarity concerning the efficacy of pharmaceutical treatment for NAFLD. The ability to accurately predict the disease progression and genetic/epigenetic risk factors associated with NAFLD will help to target specific, effective treatments for each patient [56]. Prospective studies in the future ought to include a sizable sample size, a suitable design, and a lengthy patient follow-up. Upcoming research initiatives that combine patient epigenetic, transcriptomic, proteomic, and metabolomic data with health records, preferably electronic ones, will alter the capacity to control or cure the disease [57]. To better understand the underlying biological mechanisms of prevention or therapy as well as potential side effects, additional monotherapy clinical studies and pharmacological investigations are still needed in this regard. This could also be used to determine the ideal daily fucoxanthin intake for both pediatric and adult patients.

## 4. Conclusions

**Figure 1:**
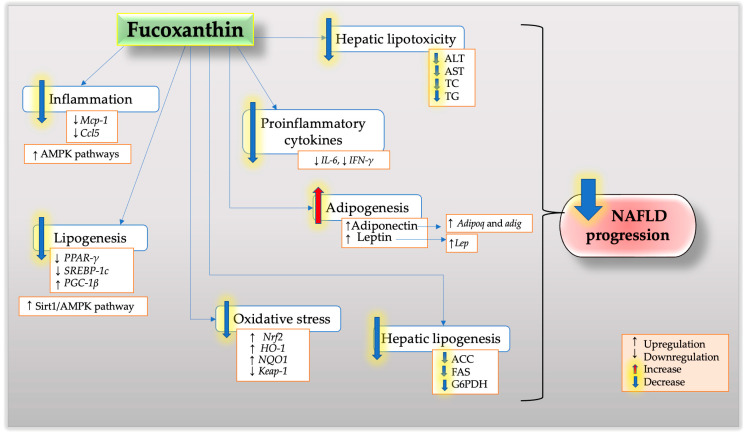
The potential effects and mechanisms of fucoxanthin on NAFLD.

This review highlights the potential beneficial effect of fucoxanthin as a naturally active compound in the treatment of NAFLD. The published findings described in the present review suggest that there is scientific evidence supporting the positive effects of fucoxanthin in NAFLD. The potential therapeutic target of fucoxanthin in NAFLD is illustrated in Figure 1. Fucoxanthin regulates numerous molecular and cellular processes in liver disease, as demonstrated by clinical trials, animal research, and cell experiments. Its potent effects on the pathogenesis of NAFLD suggest the potential that it could serve as an effective anti-steatosis, anti-inflammatory, and anti-fibrosis agent in NAFLD based on prior research involving human trials, animals, and *in vitro* research. Finally, a deeper comprehension of the pathophysiology of NAFLD will open the door to the development of combination therapy trials with high rates of therapeutic success.

## Data Availability

Not applicable.
